# Supervised and Unsupervised AI-Driven Structural Health Monitoring Framework for Additively Manufactured Metal Components

**DOI:** 10.3390/s26113547

**Published:** 2026-06-03

**Authors:** Romaine Byfield, Ahmed Shabaka, Ibrahim Tansel

**Affiliations:** Department of Mechanical and Materials Engineering, Florida International University, Miami, FL 33147, USA; ashab014@fiu.edu (A.S.); tanseli@fiu.edu (I.T.)

**Keywords:** structural health monitoring, piezoelectric transducers, signal processing, time–frequency analysis, machine learning, deep learning, supervised learning, unsupervised learning, convolutional neural networks, Gaussian mixture model

## Abstract

Structural health monitoring (SHM) of additively manufactured (AM) small and complex components is investigated using a sensor-based signal processing and machine-learning framework. Guided-wave responses acquired from piezoelectric transducers are analyzed to evaluate the performance of sweep-sine and pulse excitation signals, as well as the influence of infill patterns, part geometry, and defect type on system reliability. Test specimens, including dogbone structures and a simulated rocket-nozzle component, were fabricated using AM, and nonstationary guided-wave signals were recorded and processed. Time–frequency signal representations (scalograms) were generated using the Continuous Wavelet Transform (CWT). Convolutional Neural Networks (CNNs) and Gaussian Mixture Models (GMMs) were employed for supervised classification and unsupervised clustering, respectively. Sweep-sine excitation consistently yielded higher classification accuracy, with CNN analysis achieving near-perfect performance and GMM clustering demonstrating improved group separability. In contrast, pulse excitation revealed transient signal features associated with wave interactions, including reflections, mode conversion, and scattering, highlighting its potential for complementary signal-based diagnostics. Importantly, the proposed hybrid supervised–unsupervised learning framework enables the quantification of previously unseen intermediate load states, demonstrating strong adaptability and generalizability beyond the conditions represented in the training data.

## 1. Introduction

The safety and reliability of engineering structures depend critically on the timely detection of damage and degradation. Failures in civil, aerospace, and mechanical systems can lead to severe economic losses and safety risks, underscoring the importance of monitoring techniques capable of providing accurate and continuous insights into structural performance. structural health monitoring (SHM) has emerged as an effective approach for evaluating structural behavior under operational conditions, enabling the identification of damage, changes in loading, and variations in material properties through measured system responses [1].

Recent advances in sensing technologies, data acquisition systems, and computational methods have significantly expanded the capabilities of SHM across a wide range of applications. These developments have enabled more reliable and data-driven assessments of structural integrity, facilitating improved maintenance strategies and predictive diagnostics in complex engineering systems.

In parallel, additive manufacturing (AM) has gained increasing adoption for the production of metallic components with complex geometries and enhanced design flexibility. Its ability to fabricate intricate features and optimize material usage has made it particularly attractive for high-performance applications in aerospace and mechanical engineering. However, their structural integrity is often lower than that of conventionally manufactured components, making the implementation of structural health monitoring (SHM) systems essential for improving the reliability of machines that incorporate AM parts [2]. Unlike the large flat plates typically examined in early SHM research, AM components are often smaller and feature complex geometries, which makes SHM implementation significantly more challenging.

Active SHM systems generate surface waves in the structure, using excitation signals such as sweep-sine waves or transient pulses, to identify the presence of damage, detect changes in loading, or observe variations in material properties through analysis of wave propagation characteristics. Sweep-sine excitation concentrates energy within a narrow frequency band that sweeps across the spectrum, reducing the influence of external noise. In contrast, pulse excitation stimulates a broad range of frequencies simultaneously, allowing hidden structural dynamics to be revealed during the non-excitation intervals between transients. Producing sweep-sine signals generally requires analog circuitry and is more expensive, whereas pulse signals can be generated easily and cost-effectively using microprocessors.

Recent advances in sensing technologies and data processing have made SHM feasible across a wide range of applications, including aircraft fuselages, wind turbine blades, and other aerospace structures. With the growing adoption of additive manufacturing, ensuring the integrity and reliability of AM metal components has become increasingly important, attracting substantial research interest [3].

### 1.1. Structural Health Monitoring

Structural health monitoring (SHM) is a data-driven discipline that evaluates the condition of structures over time through continuous or periodic measurements. Although structural observations have historically supported theoretical developments in structural mechanics [4,5], SHM extends this tradition into the modern era through intelligent sensing technologies and advanced analytical methods [4].

Recent advances in sensing and information technologies have enabled active SHM methodologies [6,7,8] capable of interpreting a structure’s dynamic characteristics and detecting abnormalities without intrusive testing. By analyzing sensor data, engineers can calibrate models with field measurements, identify damage, and predict future risks. SHM accomplishes this by integrating state-of-the-art algorithms, inverse dynamics, and real-time monitoring into a comprehensive diagnostic framework [6].

### 1.2. Sweep Sine Wave Excitation

**Figure 1:**
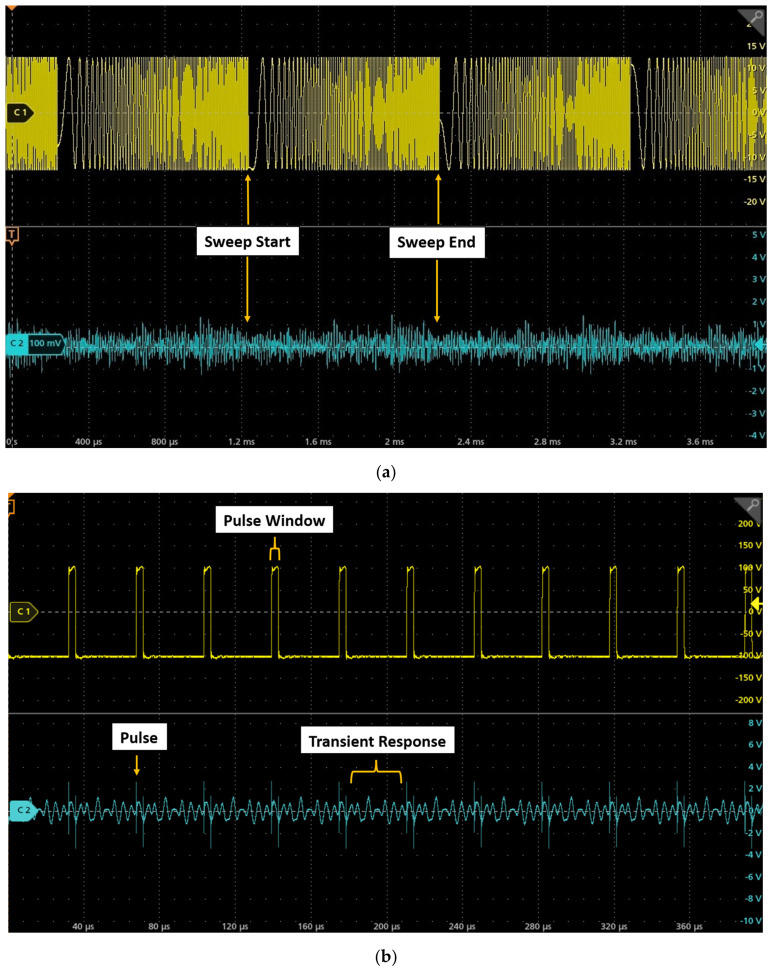
Signal and response from: (**a**) sweep sine wave; (**b**) pulse wave.

Sweep-sine wave excitation involves stimulating a structure with a signal whose frequency gradually increases or decreases over a specified range (Figure 1a). This method provides a highly controlled means of exciting the system, ensuring that energy is introduced consistently across the frequency spectrum. Sweep-sine excitation is particularly effective for identifying frequency-dependent behavior, resonance, and modal characteristics of structures [9], even in environments with significant external noise.

In SHM applications, sweep-sine excitation is commonly used to assess the global dynamic response of a structure. Its predictability and smooth frequency transition make it well suited for system identification, baseline modeling, and the detection of large-scale damage. However, its ability to reveal subtle nonlinearities or highly localized defects may be limited, particularly in complex or heterogeneous materials [10].

### 1.3. Pulse Wave Excitation

Pulse excitation uses short-duration, high-intensity signals that introduce a broad spectrum of frequencies simultaneously (Figure 1b). When such a pulse is applied, it generates a transient response followed by a mechanical reverberation within the structure. During the intervals between pulses, the response carries valuable information about internal structural interactions, including wave reflections, mode conversions, and scattering from discontinuities. By contrast, sweep sine excitation produces a continuous response, as each cycle transitions into the next, ensuring the part is constantly excited across the frequency range.

This property makes pulse excitation particularly suitable for identifying localized damage or changes in structural boundaries. In SHM, especially when combined with advanced signal processing and machine learning techniques, pulse excitation can yield high-sensitivity diagnostics. The response characteristics during the free-propagation period allow detection of subtle changes in loading, defects, or internal geometry, making it a powerful tool for structures with complex or evolving characteristics [11,12].

### 1.4. Machine and Deep Learning

As the demand for intelligent infrastructure systems grows, structural health monitoring (SHM) has increasingly turned to machine learning to interpret complex structural response data and make informed assessments [13]. In SHM, a key area of research is the development of diagnostic algorithms capable of identifying structural damage. Such algorithms are designed to process monitoring data and provide critical information regarding whether damage has occurred, where it is located, and how severe it is [6]. Among these approaches, supervised and unsupervised learning have emerged as complementary tools capable of uncovering damage patterns, assessing system conditions, and predicting structural behavior.

Supervised learning models learn from labeled datasets where the structural condition (e.g., healthy or damaged) is known, making them ideal for classification and regression tasks. These models, when well-trained, can generalize well to new data with similar patterns. In SHM, they have been widely adopted for tasks such as crack classification, stress estimation, and damage severity assessment. For example, Kim and Mukhiddinov developed a Convolutional Neural Network (CNN)-based anomaly detection method for civil infrastructure that achieves high accuracy in time-series classification under varying sensor and anomaly classes [14].

However, unsupervised learning plays a crucial role in situations where labeled data are scarce, expensive to generate, or entirely unavailable. These methods explore the underlying structure of the data without prior knowledge of class labels, offering a powerful means of anomaly detection and pattern discovery in long-term monitoring systems. Soleimani-Babakamali et al. proposed a mixture of GANs and one-class Gaussian models for unsupervised real-time SHM, which identifies damage categories robustly even without many labels [15]. Also, autoencoder-based unsupervised methods have been used to detect anomalous behavior in bridges under environmental and external variations, showing promise in catching “outlier” or damage signatures that supervised classifiers may miss [16].

## 2. Theoretical Background

### 2.1. Supervised Learning for Structural Health Monitoring

Supervised learning has emerged as a powerful tool in structural health monitoring (SHM), particularly when labeled data representing known structural states such as healthy, damaged, or loaded conditions are available. These methods train a model to learn the mapping between sensor inputs and predefined output labels, enabling the system to classify new observations with high accuracy [17,18,19].

**Figure 2:**
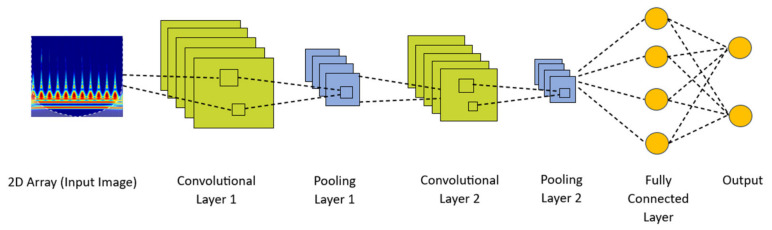
Typical 2D CNN architecture.

In this study, 2D Convolutional Neural Networks (2D-CNNs) were employed to analyze scalogram images generated using Continuous Wavelet Transform (CWT), as shown in Figure 2. As opposed to 1D-CNNs, which are particularly suited for raw time-series data collected directly from piezoelectric sensors [18,19,20], this approach enables the model to capture rich time–frequency representations of the sensor signals, which are often more sensitive to subtle structural changes compared to time-domain features alone [21,22].

The learning objective in both CNN architectures is to minimize the categorical cross-entropy loss:

(1) $$L_{CE}=-\sum_{i=1}^{N}\sum_{j=1}^{C}y_{ij}\cdot \log\left({\hat{y}}_{ij}\right)$$

where $y_{ij}$ is the ground truth label for sample i and class j, and ${\hat{y}}_{ij}$ is the predicted class probability from the SoftMax output of the CNN. *N* is the number of training samples, and *C* is the total number of classes.

The CNN architecture employed in this work consists of three convolutional blocks with 5 × 5 kernels, stride 4, and 16 filters per layer, each followed by batch normalization and ReLU activation. Average pooling layers are used for downsampling. The network terminates in a fully connected layer with the number of outputs corresponding to the number of target classes. Batch normalization, together with a computationally efficient architecture, effectively mitigates overfitting, reducing the need for dropout.

For optimization, the training process uses Stochastic Gradient Descent with Momentum (SGDM), which enhances convergence by incorporating the previous update direction:

(2) $$v_{t}=\mu v_{t-1}-\eta \nabla L\left(\theta_{t}\right),\;\;\theta_{t+1}=\theta_{t+1}+v_{t}$$

where μ is the momentum coefficient, η is the learning rate, θ represents the model parameters, and $v_{t}$ is the update vector.

Supervised models offer high classification performance when trained on well-labeled datasets, allowing the CNNs to distinguish between subtle variations in wave signals caused by different damage types, loading conditions, or material heterogeneity. In this study, 2D Convolutional Neural Networks (2D-CNNs) were utilized to exploit deeper feature hierarchies in spectro-temporal data, enabling the extraction of rich time–frequency characteristics that improve sensitivity to subtle structural changes [17,19,20,22].

Nevertheless, supervised learning in SHM is inherently constrained by its dependence on labeled data. These models may struggle when encountering previously unseen structural states, such as unexpected defects or loading scenarios not represented in the training set [18,20].

When used in a hybrid learning framework, supervised CNNs offer rapid, accurate identification of known structural states, while unsupervised models support adaptive monitoring by detecting anomalous patterns. Together, they form a robust classification pipeline for real-time SHM systems [17,23,24].

### 2.2. Unsupervised Learning for Structural Health Monitoring

In many real-world SHM applications, the lack of labeled data poses a significant challenge, particularly as structural conditions evolve over time or deviate from known failure modes [16,25,26]. To address this, unsupervised learning techniques provide a means to extract meaningful patterns directly from raw data. One such approach, clustering using the Gaussian Mixture Model (GMM), offers a probabilistic framework for identifying underlying structural states and detecting anomalies without predefined labels [8,23,27]. Early reviews of SHM methods highlighted the importance of unsupervised learning for cases where damage signatures are not predefined [8], while more recent work has demonstrated the effectiveness of GMM combined with expectation–maximization for probabilistic damage detection [27].

GMM assumes that observed data are generated from a combination of multiple Gaussian distributions, each representing a potential structural state. The model is defined by the probability density function [28]:

(3) $$p\left(x\right)=\sum_{k=1}^{K}\pi_{k}\cdot N\left(x|\mu_{k},\sum_{k}\right)$$

where π_k_ are the mixture weights, µ_k_ and ∑_k_ represent the mean and covariance of each Gaussian component, and K is the number of clusters.

**Figure 3:**
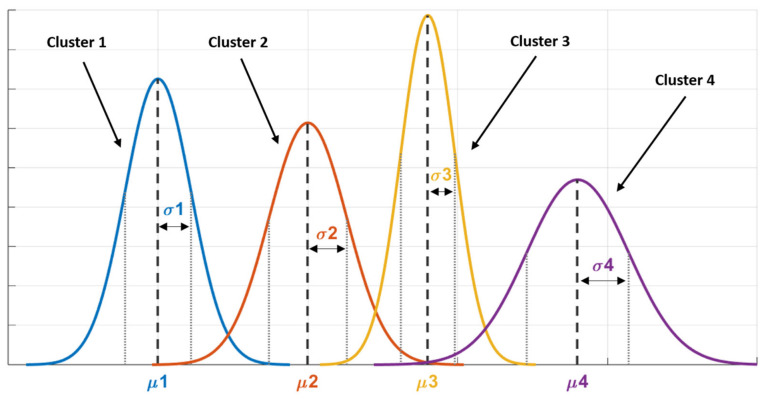
A Gaussian normal distribution plot showing each cluster.

In a Gaussian Mixture Model, each bell curve represents a probability distribution that describes one cluster of data (Figure 3). The x-axis corresponds to the feature values, where the mean (μ) marks the center of the cluster and the standard deviation (σ) defines how widely the points are spread around that center. In a scatter plot, each data point is assigned to the cluster whose Gaussian distribution gives it the highest probability, meaning the curves provide a statistical boundary that explains why a point belongs to one cluster over another [29,30].

To estimate the parameters (πk, µk, ∑k), the Expectation-Maximization (EM) algorithm is employed. This iterative method alternates between calculating the expectation (E-step), which assigns soft labels to the data, and maximization (M-step), which updates the parameters to best fit the data [31,32].

In this study, a full covariance structure was selected for the Gaussian components. Unlike spherical or diagonal covariances, which constrain clusters to circular or axis-aligned ellipsoidal shapes, full covariance allows clusters to assume arbitrary orientations in feature space. This flexibility is critical in SHM applications, where structural response data may exhibit correlated features and anisotropic spread due to complex loading conditions or evolving damage states. Using a full covariance matrix improves the model’s ability to capture elongated or rotated cluster shapes, thereby enhancing classification accuracy and adaptability [29,32].

A critical component of the framework proposed in this paper is the dynamic determination of the number of clusters (K). Rather than predefining K, the system uses the Bayesian Information Criterion (BIC) to evaluate models with varying numbers of components, selecting the one that balances model complexity and data fit [33,34]:

(4) $$BIC\;=\;-2log\cdot \left(L\right)+k\cdot log\left(n\right)$$

where L is the maximized value of the likelihood function of the model, k is the number of parameters in the model, and n is the number of data points.

The iterative fitting of the model to new incoming data enables adaptive learning, allowing the system to autonomously detect new structural states or evolving damage signatures without prior labels [16,35]. This capability makes GMM particularly suited for real-time monitoring applications, such as detecting unforeseen anomalies in critical infrastructure [25,30]. When integrated into a live SHM system, this approach allows for continuous updating and re-clustering as conditions evolve, thereby enhancing the system’s long-term reliability and responsiveness.

Through the combination of GMM’s probabilistic flexibility and the ability to optimize model complexity using BIC, the presented unsupervised framework addresses a key limitation in traditional SHM methods: the lack of scalable and autonomous classification tools for unlabeled or evolving structural conditions [32,35].

### 2.3. Integrating Deep Learning and Probabilistic Clustering for Real-Time SHM

In a live monitoring system, the integration of CNNs and GMM enables a hybrid approach to SHM, where CNNs provide rapid classification of known damage types and GMM contributes probabilistic clustering for unforeseen conditions. Complementary approaches such as transfer learning further enhance this framework by allowing CNN models to adapt to new structural scenarios with limited retraining [36,37]. Meanwhile, the GMM-based clustering method enables early detection of unknown damage patterns, offering a proactive approach to structural safety [38,39]. As the structure undergoes changes due to environmental factors or operational loads, the clustering model adapts, ensuring that new damage states are not overlooked. This adaptability makes the approach well-suited for long-term monitoring of critical infrastructure, such as bridges, aircraft, and offshore platforms, where evolving damage mechanisms must be continuously assessed [37,38,40].

By leveraging both deep learning paradigms, this study presents a comprehensive framework that enhances the reliability and adaptability of SHM systems. The combination of supervised and unsupervised learning ensures that both known and unknown damage states are effectively detected, paving the way for more intelligent and autonomous SHM solutions.

Compared to traditional threshold-based or single-sensor monitoring approaches, the proposed hybrid CNN–GMM framework offers several key advantages. It enables automated classification of multiple structural conditions, allowing the system to distinguish subtle variations in sensor signals caused by different damage types or loading scenarios [41,42]. The GMM-based clustering further provides the ability to detect previously unseen anomalies and adaptively update cluster assignments as structural states evolve over time, enhancing long-term monitoring reliability [43,44]. By integrating multi-modal features, such as time-series signals and scalogram representations, this approach also captures complex correlations that conventional single-channel analyses may overlook, reducing the need for manual interpretation and improving overall diagnostic sensitivity [36,45,46]. Collectively, these capabilities position the hybrid framework as a more intelligent and resilient SHM solution for dynamic, real-world infrastructure systems.

## 3. Materials and Methods

This study involved two primary experiments designed to evaluate the response of additively manufactured stainless steel specimens under varying structural and loading conditions using both sweep sine and pulse excitations. All test samples were fabricated using the Metal X system (Markforged, Waltham, MA, USA), which employs a process known as Atomic Diffusion Additive Manufacturing (ADAM). In this process, a filament composed of metal powder bound within a polymer matrix is first extruded layer-by-layer to form a “green” part. The printed component then undergoes a debinding stage, during which the polymer binder is removed, leaving behind a fragile “brown” part consisting primarily of metal particles. This is followed by a high-temperature sintering process, where the metal particles diffuse and densify to produce a fully metallic component [47].

**Figure 4:**
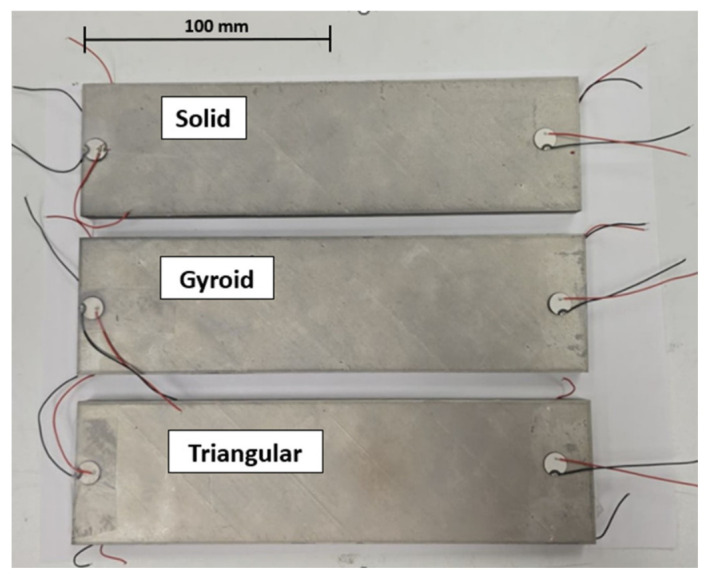
Rectangular metallic bar specimens with varying infills.

The first experiment involved three rectangular stainless-steel bars (Figure 4) with dimensions of 200 mm × 60 mm × 10 mm, each manufactured with a distinct internal structure: one solid (fully dense), one with a triangular infill pattern composed of repeating triangular cells, and one with a gyroid infill consisting of a continuous periodic lattice structure. These variations allowed the study to investigate how internal geometry influences signal propagation. For each bar, piezoelectric (PZT) disk transducers were bonded at opposite ends, one to serve as the actuator and the other as the receiver. Excitation signals in the form of a sweep sine wave ranging from 100 kHz to 300 kHz over 1 ms and amplitude of 20 Vpp, and a pulse signal with a frequency of 28 kHz and amplitude of 20 Vpp, were sequentially applied. The receiving PZT captured the resulting waveform, which was then transmitted to a 2 Series oscilloscope (Tektronix, Inc., Beaverton, OR, USA) and stored for subsequent analysis.

**Figure 5:**
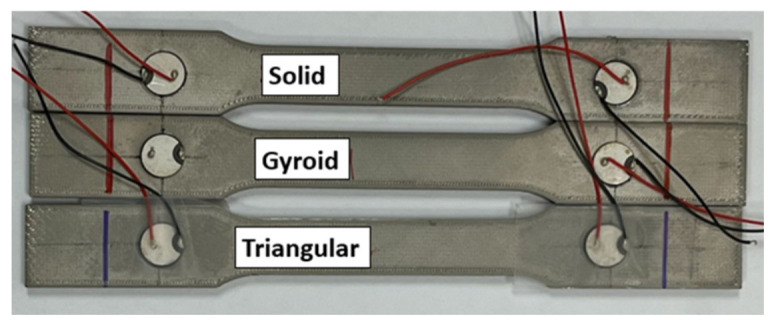
Tensile test (dogbone) specimens based on ASTM D638.

**Figure 6:**
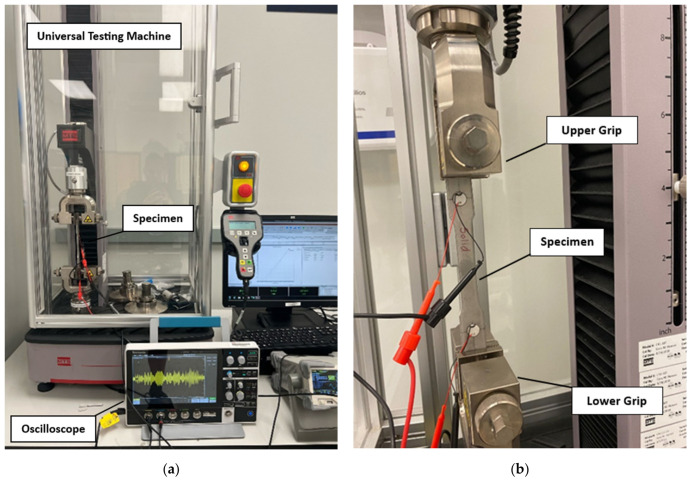
(**a**) Experimental setup for the tensile force test; (**b**) Close-up of tensile test specimen mounted in the UTM with probes attached.

The second experiment focused on additively manufactured dogbone specimens, also composed of stainless steel with solid, triangular, and gyroid infill configurations (Figure 5). Similar to the first setup, PZT disks were affixed to both ends of each specimen. The dogbones were mounted in a universal testing machine (UTM) and subjected to tensile loads of up to 900 N, applied incrementally at 100 N intervals. The tests stopped at 900 N in order to stay within the limits of the UTM, which is 1000 N. As the specimens were stressed, excitation signals were again delivered via the PZT actuator, and the resulting response was collected by the receiving PZT and recorded by the oscilloscope. Figure 6 illustrates the experimental setup used for this test.

**Figure 7:**
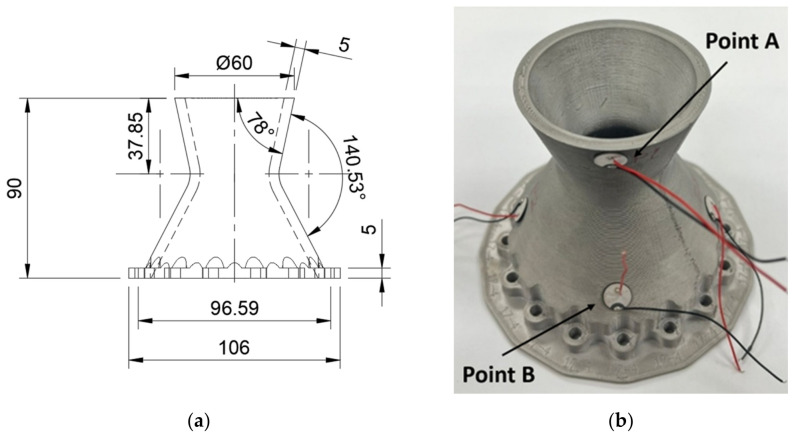
(**a**) Rocket nozzle dimensions in mm; (**b**) 3D-printed rocket nozzle.

**Figure 8:**
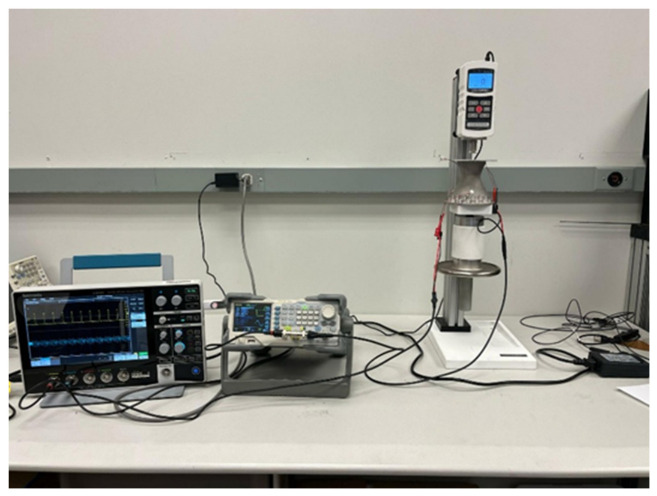
Experimental setup for compressive force test.

The third experiment utilized a 3D-printed simulated rocket nozzle specimen (Figure 7a) mounted on a Mark-10 test stand equipped with a Mark-10 force gauge. As shown in Figure 7b, piezoelectric transducers (PZTs) were attached at two locations on the nozzle: point A and point B. The PZT at point A served as the actuator, delivering excitation signals, while the PZT at point B functioned as the sensor, capturing the structural response. The additively manufactured rocket nozzle served as a representative complex engineering structure demonstrating the applicability of the approach to a more realistic engineering component with complex geometry. Figure 8 shows the complete experimental setup for this test. Compressive forces were applied incrementally to the nozzle, starting from 0 N up to 1000 N in steps of 100 N. The resulting response signals were collected in the same manner as in the previous experiments for subsequent analysis.

**Figure 9:**
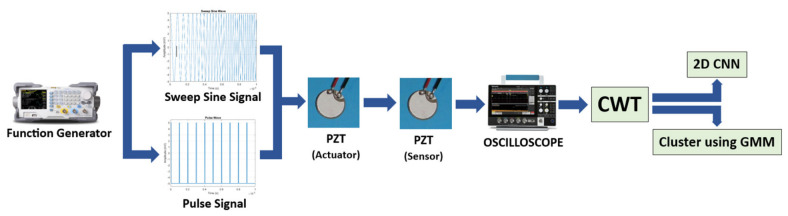
Experimental workflow.

As illustrated in Figure 9, all collected time-domain signals were processed in MATLAB (R2024b) using Continuous Wavelet Transform (CWT) to generate time–frequency scalograms, which served as inputs for subsequent machine learning analysis. A 2D-CNN was trained on the scalograms for supervised classification of structural conditions.

In parallel, GMM clustering was employed in an unsupervised manner to classify structural states in the absence of predefined labels. An incremental Gaussian Mixture Model (GMM) clustering approach was employed, where each CWT scalogram image was represented using two statistical features: (1) the mean pixel intensity, representing the overall energy distribution of the time–frequency representation, and (2) the standard deviation of pixel intensity, representing the variability or dispersion of energy within the scalogram. These two features form a two-dimensional feature space that is directly used as input to the GMM. Therefore, the observed clustering compactness reflects the similarity of signals in terms of their global energy distribution and variability within the time–frequency domain. The clustering model was updated sequentially as new datasets were introduced, enabling adaptive refinement of cluster boundaries. The optimal number of clusters was determined using the BIC at each stage. Figure 9 provides a schematic overview of the experimental approach and the SHM data acquisition and processing framework used in this study.

**Figure 10:**
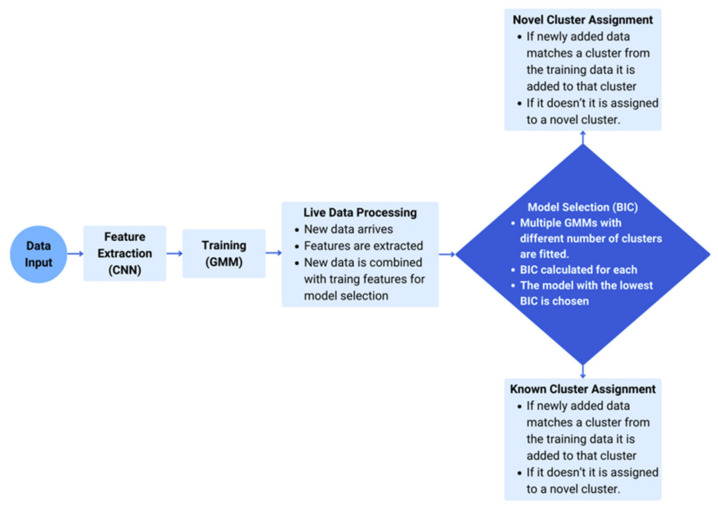
Hybrid approach to a SHM system using both 2D CNN and GMM.

In addition to these approaches, a hybrid method represented in Figure 10 was implemented in which the 2D CNN served as a feature extractor, providing high-level representations of the input scalograms. Rather than using the CNN exclusively for classification, the extracted feature vectors were used as inputs to the GMM. To enable visualization, principal component analysis (PCA) was applied to project the feature space onto two dimensions, where the horizontal and vertical axes correspond to the first and second principal components, respectively. This allowed the GMM to perform clustering in a feature space enriched with discriminative patterns learned by the CNN, improving its ability to separate structural conditions. The first principal component captures the direction of maximum variance in the CNN-derived feature space, representing the most dominant variation in the learned time–frequency patterns, while the second principal component captures the next most significant orthogonal variation. These components are linear combinations of high-dimensional CNN features and reflect dominant data-driven variations associated with different structural conditions.

Again, the BIC was employed to determine the optimal number of clusters and to prevent overfitting. This hybrid strategy combined the deep feature-learning capability of CNNs with the probabilistic adaptability of GMMs, making it well-suited for scenarios involving evolving or previously unseen structural states.

Finally, for applied load conditions, Inverse Distance Weighting (IDW) interpolation (Equations (5) and (6)) was used to estimate the data corresponding to previously unforeseen classes. In this approach, IDW assigns weights to nearby training samples inversely proportional to their distance from the new data point, enabling interpolation of the load values for samples that do not belong to any of the predefined training classes.

(5) $$z\left(x\right)=\frac{\sum_{i=1}^{n}w_{i}z_{i}}{\sum_{i=1}^{n}w_{i}},$$

(6) $$w_{i}=\frac{1}{d_{i}^{p}}$$

where *z*(*x*) is the estimated value at the location *x*, *z_i_* is the known value at point *i*, *n* is the number of known points, *w_i_* is the calculated weight, and *d_i_* is the distance between the known and unknown points.

## 4. Results

**Figure 11:**
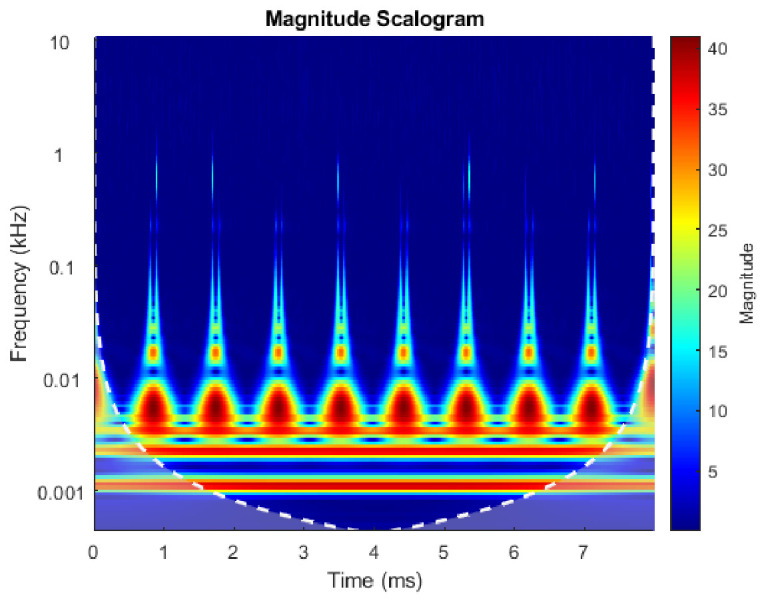
CWT scalogram showing the signal’s time–frequency content, with time (ms) on the x-axis, frequency (MHz) on the y-axis, and color indicating magnitude (warmer colors represent higher energy). The dashed line curve denotes the cone of influence, beyond which boundary effects may affect reliability of wavelet coefficients.

**Figure 12:**
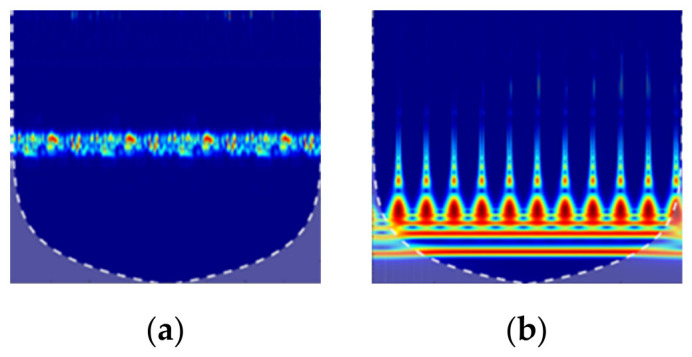
(**a**) Scalogram created by sweep sine wave excitation of the solid dogbone specimen; (**b**) Scalogram created by pulse wave excitation of the solid dogbone specimen. Axis labels are intentionally omitted, as these images are presented in the same format used for training and classification by the learning algorithms.

Figure 11 and Figure 12 show the resulting scalograms after the raw data signals are processed using the CWT. Figure 12 shows a stark difference in the signal intensity across the frequency range.

### 4.1. Infill Classification (Supervised Learning—CNN)

**Figure 13:**
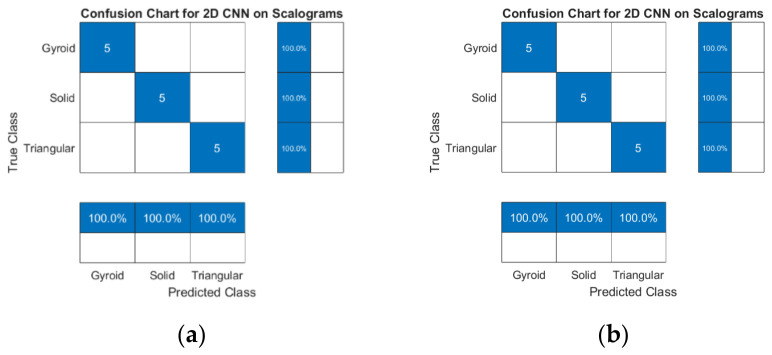
Confusion charts showing infill classification accuracy using 2D CNN: (**a**) sweep sine wave: 100% accuracy; (**b**) pulse excitation: 100% accuracy.

The proposed 2D CNN achieved excellent performance in classifying infill types under both sweep sine and pulse excitation. As shown in Figure 13, the confusion charts demonstrate 100% accuracy in distinguishing between solid, gyroid, and triangular infills. This indicates that the excitation–response signals carry unique vibrational fingerprints that are directly linked to differences in internal geometry.

### 4.2. Tensile Force Classification (Supervised Learning—CNN)

**Figure 14:**
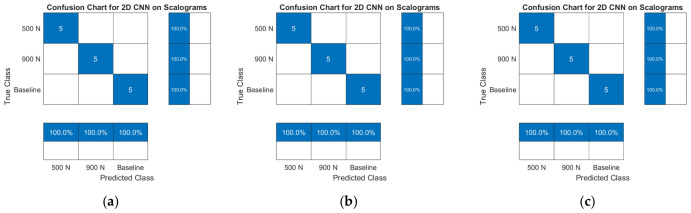
Confusion charts showing tensile test classification results for 3 classes using data from sine wave excitation: (**a**) Solid: 100% accuracy; (**b**) Gyroid: 100% accuracy; (**c**) Triangular: 100% accuracy.

**Figure 15:**
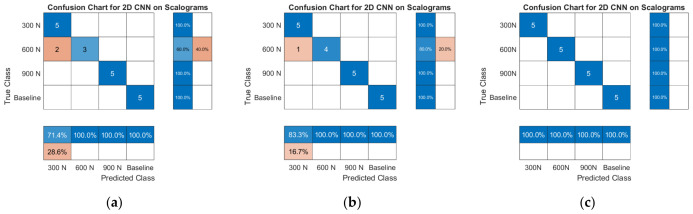
Confusion charts showing tensile test classification results for 4 classes using data from sine wave excitation: (**a**) Solid: 90% accuracy; (**b**) Gyroid: 95% accuracy; (**c**) Triangular: 100% accuracy.

**Figure 16:**
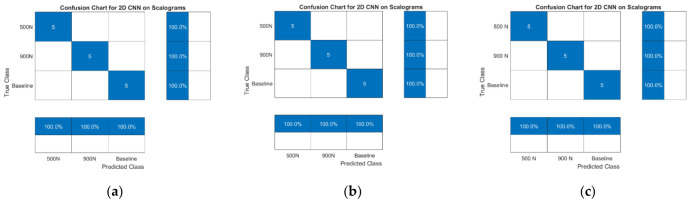
Confusion charts showing tensile test classification results for 3 classes using data from pulse wave excitation: (**a**) Solid: 100% accuracy; (**b**) Gyroid: 100% accuracy; (**c**) Triangular: 100% accuracy.

**Figure 17:**
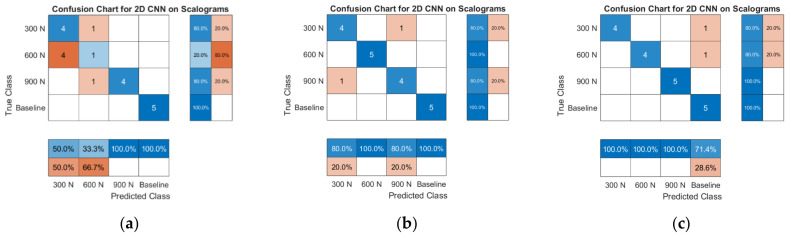
Confusion charts showing tensile test classification results for 4 classes using data pulse wave excitation: (**a**) Solid: 70% accuracy; (**b**) Gyroid: 90% accuracy; (**c**) Triangular: 90% accuracy.

Under sweep sine excitation, the CNN achieved perfect accuracy in the three-class problem (0 N, 500 N, and 900 N) across all infill types (Figure 14). In the more challenging four-class case (0 N, 300 N, 600 N, 900 N), classification remained strong: 100% for triangular, 95% for gyroid, and 90% for solid (Figure 15). For pulse excitation, the three-class scenario again produced 100% accuracy (Figure 16), while performance declined slightly in the four-class case (Figure 17), with accuracies of 90% for triangular, 90% for gyroid, and 70% for solid. Table 1 summarizes the results for all tests.

### 4.3. Compressive Force Classification (Supervised Learning—CNN)

**Figure 18:**
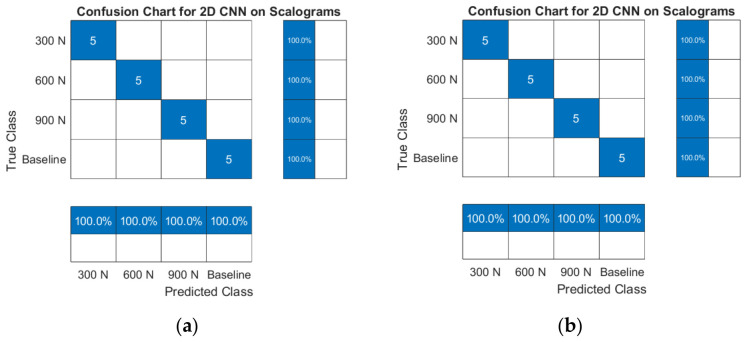
Confusion charts showing compressive force classification accuracy for 4 classes using 2D CNN: (**a**) sweep sine wave: 100% accuracy; (**b**) pulse excitation: 100% accuracy.

**Figure 19:**
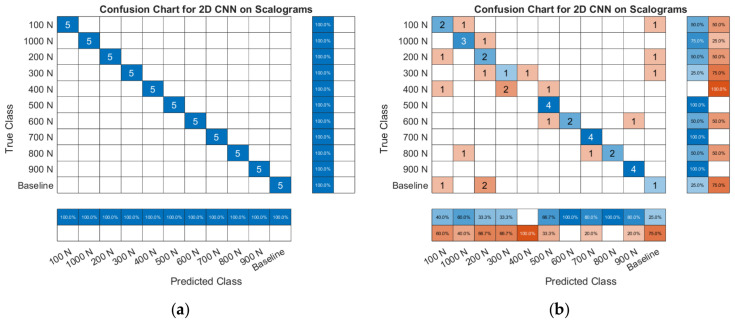
Confusion charts showing compressive force classification accuracy for 11 classes using 2D CNN: (**a**) sweep sine wave: 100% accuracy; (**b**) pulse excitation: 65.45% accuracy.

For compressive force classification on the nozzle specimen, the CNN achieved 100% accuracy in the four-class problem (0 N, 300 N, 600 N, 900 N) under both excitation types (Figure 18). When extended to eleven classes (0 N to 1000 N in 100 N increments), results diverged: sweep sine excitation maintained 100% accuracy (Figure 19), while pulse excitation dropped to 65.45%. Table 2 summarizes the results for all tests.

### 4.4. Infill Classification (Unsupervised Learning—GMM)

**Figure 20:**
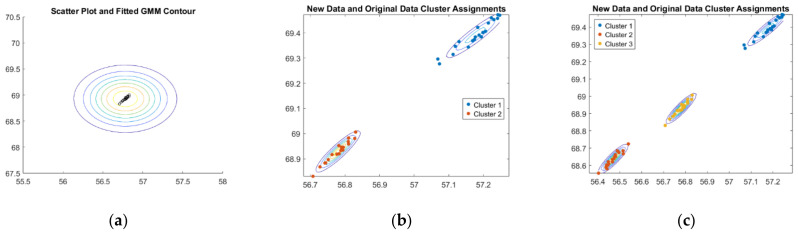
GMM cluster plots for rectangular specimens excited with a sweep wave: (**a**) Solid; (**b**) Solid and gyroid; (**c**) Solid, gyroid and rectangular.

**Figure 21:**
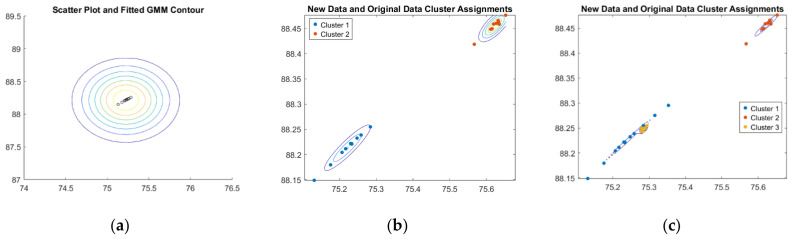
GMM cluster plots for rectangular specimens excited with a pulse wave: (**a**) Solid; (**b**) Solid and gyroid: 90% accuracy; (**c**) Solid, gyroid and rectangular.

The GMM results demonstrated consistent identification of infill types under both excitation signals (Figure 20 and Figure 21). Sweep sine excitation produced more compact and distinct clusters, while pulse excitation resulted in slightly wider spreads. Triangular infill exhibited the largest variability, solid the least, and gyroid was intermediate.

### 4.5. Tensile Force Classification (Unsupervised Learning—GMM)

**Figure 22:**
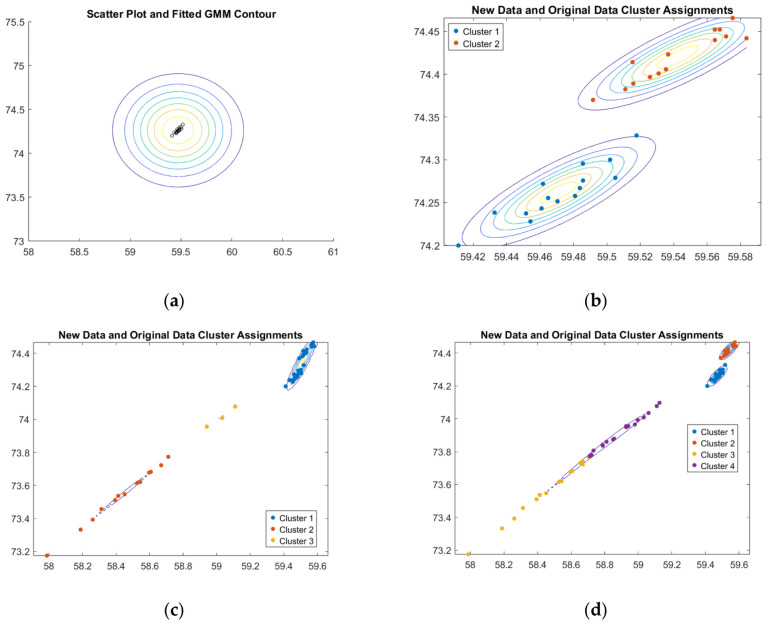
GMM cluster plots for solid infill dogbone specimen subjected to tensile loads, excited with a sweep sine wave: (**a**) Baseline; (**b**) Baseline and 300 N; (**c**) Baseline, 300 N and 600 N; (**d**) Baseline, 300 N, 600 N and 900 N.

**Figure 23:**
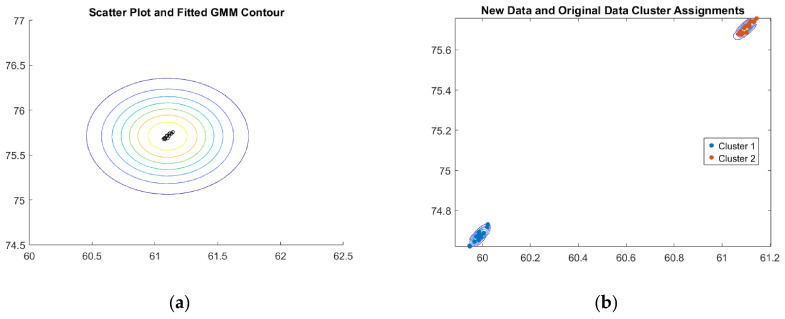

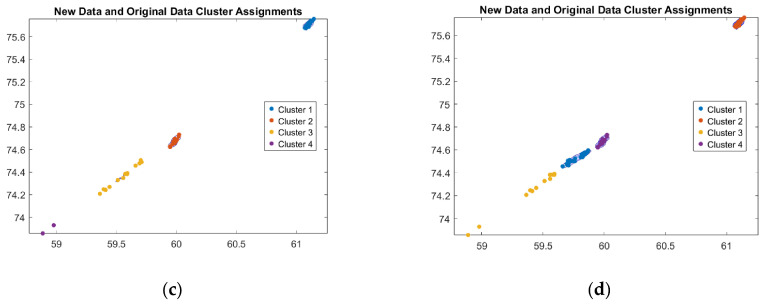
GMM cluster plots for gyroid infill dogbone specimen subjected to tensile loads, excited with a sweep sine wave: (**a**) Baseline; (**b**) Baseline and 300 N; (**c**) Baseline, 300 N and 600 N; (**d**) Baseline, 300 N, 600 N and 900 N.

**Figure 24:**
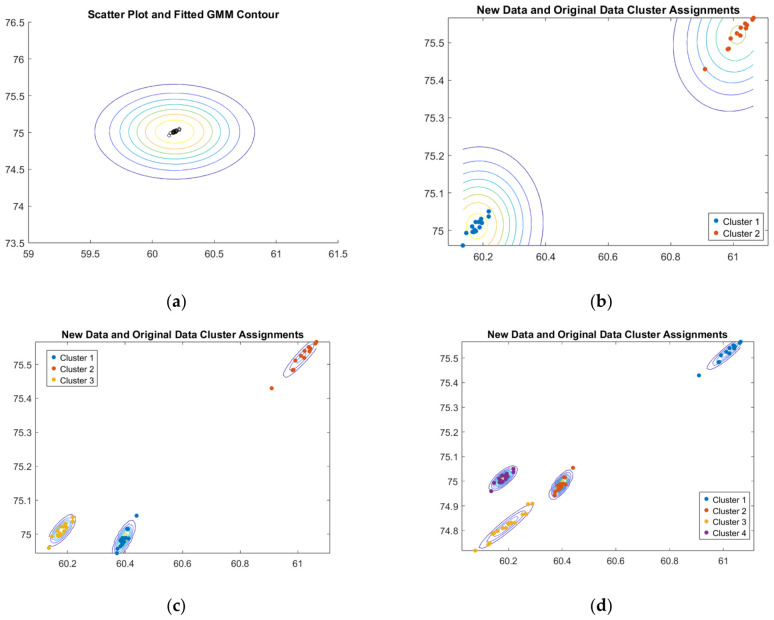
GMM cluster plots for a triangular infill dogbone specimen subjected to tensile loads, excited with a sweep sine wave: (**a**) Baseline; (**b**) Baseline and 300 N; (**c**) Baseline, 300 N and 600 N; (**d**) Baseline, 300 N, 600 N and 900 N.

**Figure 25:**
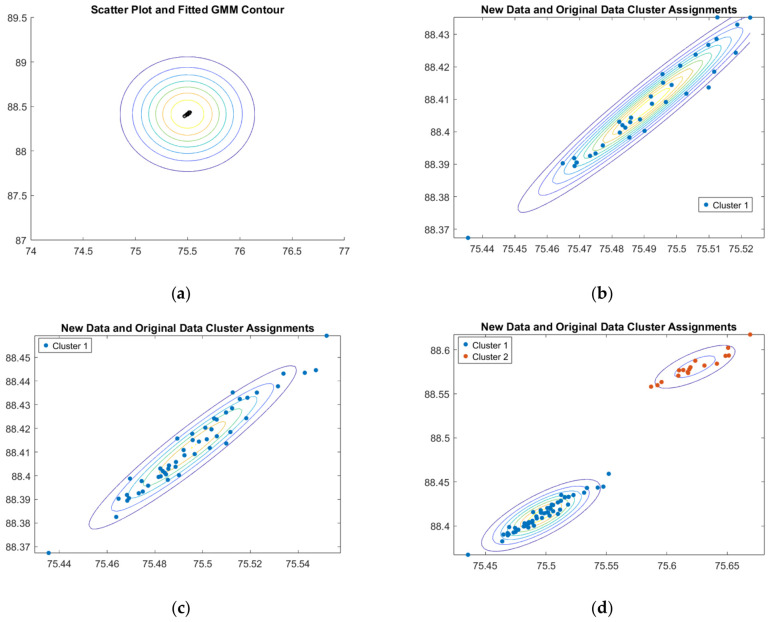
GMM cluster plots for solid infill dogbone specimen subjected to tensile loads, excited with a pulse wave: (**a**) Baseline; (**b**) Baseline and 300 N; (**c**) Baseline, 300 N and 600 N; (**d**) Baseline, 300 N, 600 N and 900 N.

**Figure 26:**
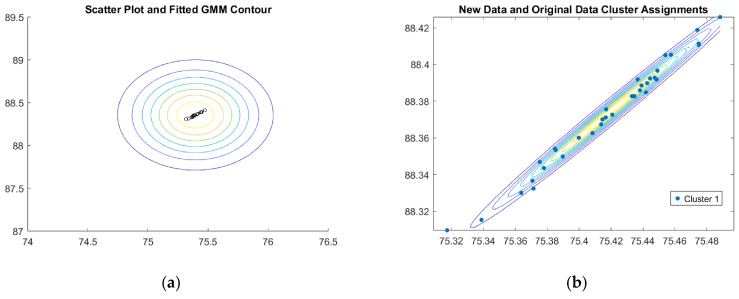

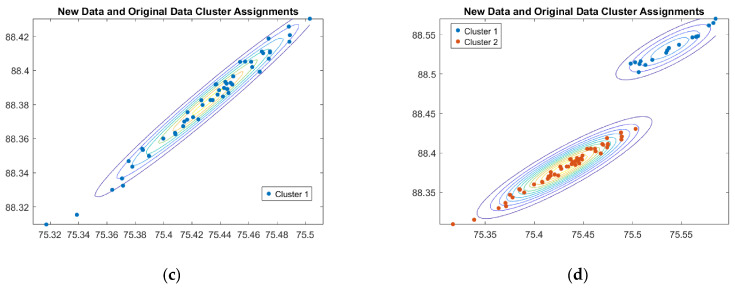
GMM cluster plots for gyroid infill dogbone specimen subjected to tensile loads, excited with a pulse wave: (**a**) Baseline; (**b**) Baseline and 300 N; (**c**) Baseline, 300 N and 600 N; (**d**) Baseline, 300 N, 600 N and 900 N.

**Figure 27:**
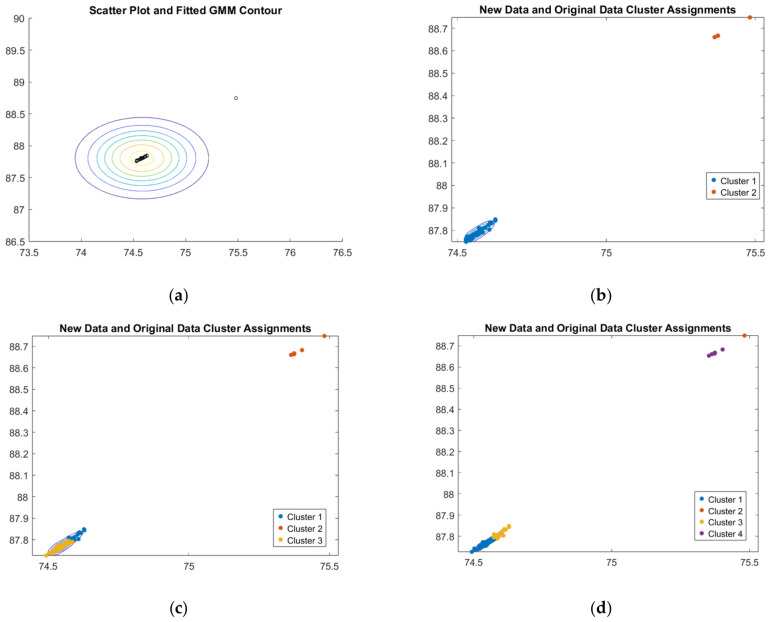
GMM cluster plots for triangular infill dogbone specimen subjected to tensile loads, excited with a pulse wave: (**a**) Baseline; (**b**) Baseline and 300 N; (**c**) Baseline, 300 N and 600 N; (**d**) Baseline, 300 N, 600 N and 900 N.

With sweep sine excitation, GMM clustering revealed distinct groupings for all load levels across infill types (Figure 22, Figure 23 and Figure 24). The triangular infill specimen’s clusters were best separated, while gyroid and solid displayed overlaps at intermediate loads. With pulse excitation, distinct clusters appeared only at the highest load (900 N) for the solid and gyroid specimens (Figure 25, Figure 26 and Figure 27), while the specimen with triangular infill maintained separation across all load levels, though not as distinct as the plot attained using sweep sine wave excitation.

### 4.6. Compressive Force Classification (Unsupervised Learning—GMM)

**Figure 28:**
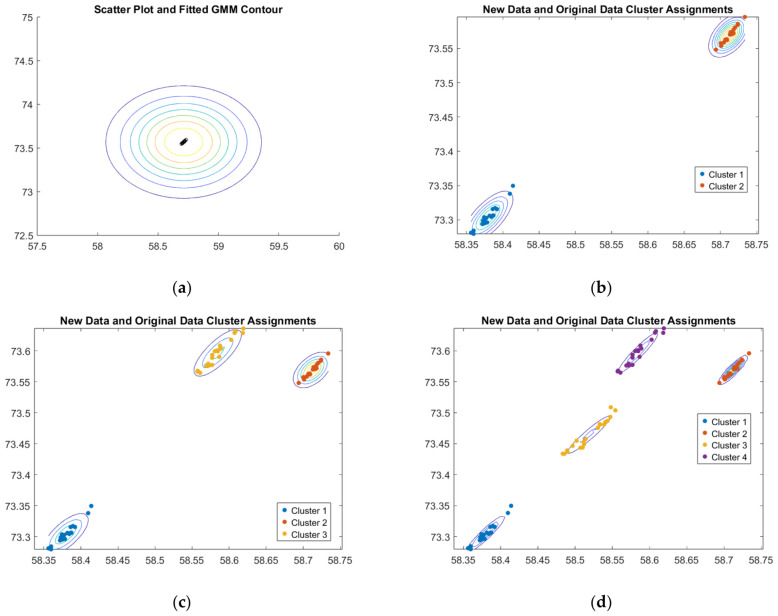
GMM cluster plots for a nozzle subjected to compressive loads, excited with a sweep sine wave: (**a**) Baseline; (**b**) Baseline and 300 N; (**c**) Baseline, 300 N and 600 N; (**d**) Baseline, 300 N, 600 N and 900 N.

For compressive force classification, sweep sine excitation again produced clearly separated and well-defined clusters across all load levels, as shown in Figure 28. The distinct separation indicates that the continuous frequency sweep effectively captured the progressive changes in structural response as compressive forces increased, allowing the Gaussian Mixture Model (GMM) to group data points into meaningful and stable clusters. This can be attributed to the broadband nature of the sweep excitation, which excites a wide range of frequencies and wave modes, making it more sensitive to stress-induced variations in wave propagation characteristics such as velocity and dispersion.

**Figure 29:**
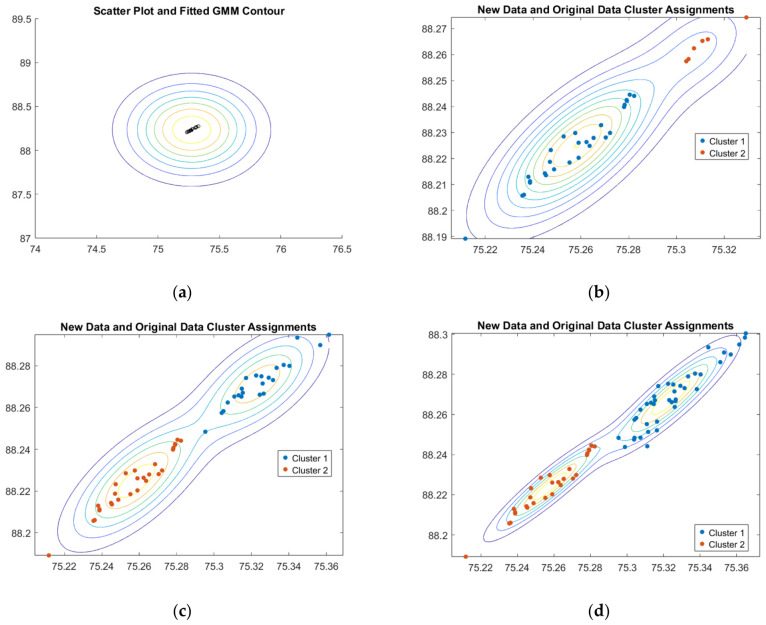
GMM cluster plots for a nozzle subjected to compressive loads, excited with a pulse wave: (**a**) Baseline; (**b**) Baseline and 300 N; (**c**) Baseline, 300 N and 600 N; (**d**) Baseline, 300 N, 600 N and 900 N.

By contrast, under pulse excitation, clustering performance was more limited. As illustrated in Figure 29, the model was only able to distinguish between the broad categories of “no force” and “force applied,” without providing reliable separation among the intermediate load magnitudes. The close proximity and overlap of clusters in this case can be explained by the relatively narrow frequency content of the pulse signal. Because stress-induced changes in wave velocity and dispersion are often subtle, particularly under compressive loading, a narrowband or transient excitation may not sufficiently capture these variations. As a result, the corresponding time–frequency representations exhibit limited sensitivity to incremental changes in load, leading to reduced separability in the feature space.

**Figure 30:**
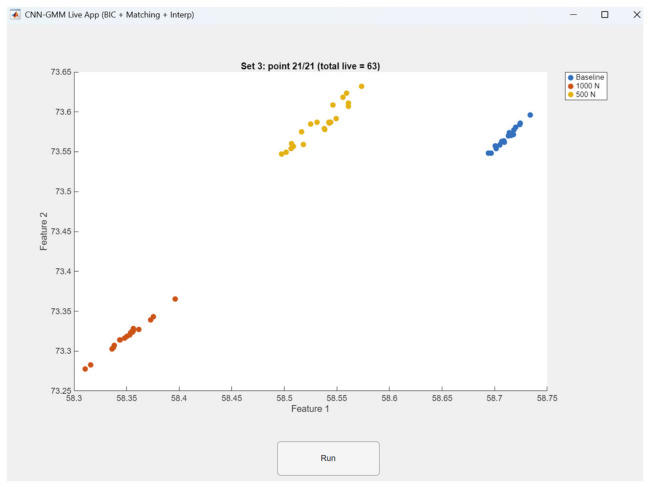
Screenshot from a live classification app classifying compressive load. The model was trained on Baseline (0 N) and 1000 N. It successfully classified the intermediate load of 500 N.

In addition, live simulation using the developed application demonstrated the capability of the classification framework not only to distinguish between trained conditions but also to quantify intermediate compressive loads. Specifically, when the model was trained using only baseline (0 N) and 1000 N data, the interpolation approach successfully assigned the correct numeric value (500 N) to previously unseen data from the intermediate load state (Figure 30). This outcome highlights the adaptive strength of the inverse distance-based labeling strategy within the GMM framework, enabling the app to approximate intermediate force magnitudes even in the absence of direct training data at those levels. A demonstration of the live classification application is provided in Video S1 (Supplementary Materials).

## 5. Discussion

The findings of this study confirm the strength of combining supervised and unsupervised learning for vibration-based SHM of additively manufactured (AM) structures. Both CNN and GMM approaches produced highly reliable results under sweep sine excitation, while performance decreased for pulse excitation, particularly in intermediate load cases. This highlights the critical role of excitation design in influencing classification accuracy and cluster separability.

### 5.1. Relation to Previous Studies

The results align with and extend prior work in the field of SHM and anomaly detection. In Structural Condition Monitoring Using Deep Learning on a Metallic Part Fabricated by Additive Manufacturing [17], CNNs were shown to be effective in capturing subtle structural variations in additively manufactured specimens. The present work builds upon that foundation by not only confirming the discriminative capability of CNNs in the frequency domain but also integrating them with GMM clustering to provide a dual supervised–unsupervised framework.

Similarly, Application of Convolutional Neural Network–Gaussian Mixture Model for Ambient Vibration-Based Structural Damage Detection by Mehboob Rasul [41] demonstrated the benefits of combining CNN feature extraction with GMM clustering for structural damage assessment. In that work, however, the CNN functioned as the primary classifier, while the GMM was used mainly to illustrate feature separability in the latent space. The present study advances this framework by employing the GMM directly as a classification tool, enabling it not only to form clusters but also to classify both known and previously unforeseen load states. Furthermore, whereas Rasul’s study focused primarily on binary damage classification (healthy vs. damaged), the current work addresses the more complex challenge of graded load classification, distinguishing incremental variations in applied forces.

Related approaches such as transfer learning have also been explored as a means to adapt CNN models to new structural scenarios with limited retraining [36,37]. While effective in extending model applicability, these methods carry important limitations for SHM. Transfer learning typically requires additional training or fine-tuning whenever new structural states emerge, which can introduce latency and computational overhead during live monitoring. In contrast, the present framework avoids retraining by leveraging adaptive GMM clustering to accommodate evolving conditions in real time, enabling faster and more efficient integration of unforeseen states into the monitoring system.

In the broader anomaly detection literature, Detecting anomalies from multiple streams using Gaussian Mixture Model with dynamic updates [30] and Real-time anomaly detection in structural health monitoring using adaptive GMM clustering [35] both emphasized the adaptive potential of GMM-based clustering for evolving systems. Our approach complements these studies by demonstrating how GMM clustering, when combined with CNN-derived features, can be extended to experimental SHM in AM parts. Importantly, while those works focused primarily on dynamic updates for anomaly detection, this study introduces a unique advancement: the ability to not only detect evolving conditions but also interpolate and assign quantitative load values to previously unseen states, thus bridging anomaly detection with load characterization in real time.

### 5.2. Classification of Unforeseen Loads

A central contribution of this work is the demonstration that intermediate load levels/values not included in the training dataset can be identified and meaningfully classified using a combination of CNN feature extraction and inverse IDW interpolation. This capability directly addresses a limitation of prior CNN- or GMM-based SHM studies, which typically require that all classes of interest be present in the training data.

By enabling the classification of unforeseen loads (e.g., correctly estimating 500 N when the model was trained only on 0 N and 1000 N), this work advances the state of SHM frameworks in several important ways. First, it enhances adaptivity to real-world conditions, where structures often encounter states not explicitly represented in training data. The ability to interpolate between known classes increases robustness and reduces dependence on exhaustive labeled datasets.

It should be noted that the interpolation is performed in the CNN-derived feature space, where weights are assigned inversely proportional to the distance between cluster centroids using a nonlinear decay relationship that emphasizes nearby structural states. While this approach does not rely on direct linear interpolation in the physical load domain, it assumes a smooth relationship between structural response and its representation in the feature space. Consequently, in cases where the structural response exhibits strong nonlinearities or abrupt transitions, interpolation accuracy may be reduced. Future work will explore and compare more advanced interpolation strategies to further improve performance under nonlinear conditions. However, the current results highlight the strong potential of the proposed approach for accurately estimating intermediate structural states from limited training data.

Second, it introduces a path toward quantitative load estimation. Unlike prior studies where GMMs or CNNs provided only categorical outputs (such as healthy versus damaged states), the present approach demonstrates that machine learning models can be extended to estimate the actual magnitude of applied loads, improving interpretability for engineering decision-making.

Finally, this advancement has direct implications for SHM applications in AM structures. Given their complex internal geometries, the ability to predict intermediate states is particularly valuable. Subtle load levels may correspond to the onset of nonlinear behavior, precursor damage, or stress concentrations, all of which are critical for early intervention and ensuring structural reliability.

### 5.3. Implications for SHM of AM Parts

The combined CNN–GMM–IDW framework represents an important step toward practical deployment of SHM systems for AM structures. CNNs provide high-confidence supervised classification where labeled data exist, while GMM clustering utilizes underlying patterns in the feature space when labels are unavailable, and IDW interpolation bridges the gap by assigning values to unforeseen intermediate states. Together, these tools form an adaptive pipeline capable of handling the uncertainty and variability inherent in AM parts.

Future work should expand this framework by extending the dataset to include a wider range of geometries, defect types, and loading scenarios, enabling broader validation of the approach. Another promising direction involves integrating multimodal sensing to further enhance both sensitivity and robustness.

By building on the strong foundations of previous CNN- and GMM-based SHM studies while introducing quantitative interpolation of unforeseen states, this work provides a pathway toward robust, adaptive, and practically deployable monitoring systems for AM structures.

## 6. Conclusions

This study investigated the potential of a combined supervised and unsupervised machine learning approach for structural health monitoring (SHM) of additively manufactured (AM) metal components. Test specimens included dogbone structures and a simulated rocket-nozzle component, as well as rectangular stainless-steel bars, all fabricated using AM. These parts featured distinct internal structures such as solid, triangular, and gyroid infill patterns. Piezoelectric transducers (PZTs) were attached to the parts to record guided-wave propagation responses. The structure was excited using two signals: a sweep sine wave (100 kHz to 300 kHz over 1 ms, 20 Vpp amplitude) and a pulse signal (28 kHz frequency, 20 Vpp amplitude). The collected time-domain signals were initially processed using the Continuous Wavelet Transform (CWT) to generate time–frequency scalograms. These scalograms served as inputs for classification: Convolutional Neural Networks (CNNs) were employed for supervised classification, and Gaussian Mixture Models (GMMs) were used for unsupervised clustering of the structural states.

The results of the study demonstrated that sweep-sine excitation consistently yielded higher classification accuracy compared to pulse excitation. In the most complex classification task—distinguishing eleven classes of compressive load (0 N to 1000 N in 100 N increments) on the nozzle specimen—the sweep sine wave excitation maintained 100% accuracy. In contrast, pulse excitation’s accuracy dropped significantly to 64.45% for the same eleven-class problem. While both excitation methods achieved 100% accuracy in simpler, four-class compressive load problems, sweep sine excitation also produced more compact and distinct clusters under GMM analysis, indicating improved group separation.

Regarding the machine learning paradigms, supervised learning (CNNs) achieved better results for the classification of known structural states, achieving near-perfect performance in several key metrics, particularly when using sweep-sine responses. For instance, CNN analysis achieved 100% accuracy in classifying infill types, three-class tensile loads, and four-class compressive loads under sweep sine excitation. Although the supervised models were highly accurate for conditions represented in the training data, the unsupervised GMM method reliably identified clusters associated with different structural states and provided the crucial capability to handle data where predefined labels were unavailable.

The proposed approach is a hybrid supervised–unsupervised framework that benefits all approaches by leveraging the strengths of both classification paradigms. This framework utilizes the 2D CNN as a feature extractor, feeding high-level representations of the scalograms into the GMM for clustering. Importantly, the hybrid framework, supported by Inverse Distance Weighting (IDW) interpolation, enabled the successful quantification of previously unseen intermediate load states. This adaptive strength was demonstrated when the model, trained only on baseline (0 N) and 1000 N data, successfully approximated the numeric value of an intermediate load state (500 N). This capability enhances the generalizability and robustness of SHM by reducing reliance on exhaustively labeled datasets covering all possible load states.

Future work will focus on refining the framework to improve the precision of detecting intermediate load values. Researchers should also concentrate on extending the methodology to other damage cases, such as varying damage sizes, and developing adaptive models that dynamically update clustering boundaries. Furthermore, future research should aim to expand the dataset to include a wider range of AM geometries, defect types, and loading scenarios, and integrate multimodal sensing to further enhance both sensitivity and robustness of the monitoring system. Ultimately, the demonstrated capability to classify and estimate previously unseen load states positions the CNN–GMM–IDW framework as a flexible and adaptive solution for next-generation SHM in AM structures.

## Figures and Tables

**Table 1:** Classification accuracy percentage for tensile loads across all infill types.

Sweep Sine Wave Excitation			
Number of Classes	Solid	Gyroid	Triangular
3	100%	100%	100%
4	90%	95%	100%
Pulse Wave Excitation			
Number of Classes	Solid	Gyroid	Triangular
3	100%	100%	100%
4	70%	90%	90%

**Table 2:** Classification accuracy percentage for compressive loads.

Number of Classes	Sweep Sine Wave Excitation	Pulse Wave Excitation
4	100%	100%
11	100%	65.45%

## Data Availability

The data supporting the findings of this study are available from the corresponding author upon reasonable request.

## Supplementary Materials

The following supporting information can be downloaded at: https://www.mdpi.com/article/10.3390/s26113547/s1, Video S1: Compressive Load Classification Demonstration Using Hybrid CNN-GMM Framework.

## Abbreviations

The following abbreviations are used in this manuscript:

AM	Additive Manufacturing/Additively Manufactured
SHM	Structural Health Monitoring
CNN	Convolutional Neural Network
CWT	Continuous Wavelet Transform
GMM	Gaussian Mixture Model
BIC	Bayesian Information Criterion
EM	Expectation-Maximization
IDW	Inverse Distance Weighting
SGDM	Stochastic Gradient Descent with Momentum
PZT	Piezoelectric Transducers
ADAM	Atomic Diffusion Additive Manufacturing
UTM	Universal Testing Machine

## References

[1] Cawley P. (2018). Structural health monitoring: Closing the gap between research and industrial deployment. Struct. Health Monit..

[2] Mower T.M., Long M.J. (2016). Mechanical behavior of additive manufactured, powder-bed laser-fused materials. Mater. Sci. Eng. A.

[3] Byfield R., Semroud G., Laurent M., Tansel I. (2023). Structural Condition Monitoring Using Deep Learning on a Metallic Part Fabricated by Additive Manufacturing. Digit. Manuf. Technol..

[4] Del Grosso A. (2013). Structural Health Monitoring: Research and Practice.

[5] Benvenuto E. (1991). An Introduction to the History of Structural Mechanics.

[6] Ozer E., Feng M. (2020). Structural Health Monitoring. Sensors and Smart Structures Technologies for Civil, Mechanical, and Aerospace Systems.

[7] Doebling S.W., Farrar C.R., Prime M.B., Shevitz D.W. (1996). Damage Identification and Health Monitoring of Structural and Mechanical Systems from Changes in Their Vibration Characteristics: A Literature Review.

[8] Sohn H., Farrar C.R., Hemez F.M., Shunk D.D., Stinemates D.W., Nadler B.R., Czarnecki J.J. (2003). A Review of Structural Health Monitoring Literature: 1996–2001.

[9] Gloth G., Sinapius M. (2004). Analysis of Swept-Sine Runs during Modal Identification. Mech. Syst. Signal Process..

[10] Rébillat M., Ege K., Gallo M., Antoni J. (2015). Repeated Exponential Sine Sweeps for the Autonomous Estimation of Nonlinearities and Bootstrap Assessment of Uncertainties. Proc. Inst. Mech. Eng. C J. Mech. Eng. Sci..

[11] Croxford A.J., Wilcox P.D., Drinkwater B.W., Konstantinidis G. (2007). Strategies for Guided-Wave Structural Health Monitoring. Proc. R. Soc. A.

[12] Lowe M.J.S., Alleyne D.N., Cawley P. (1998). Defect Detection in Pipes Using Guided Waves. Ultrasonics.

[13] Gomez-Cabrera A., Escamilla-Ambrosio P.J. (2022). Review of Machine-Learning Techniques Applied to Structural Health Monitoring Systems for Building and Bridge Structures. Appl. Sci..

[14] Kim S.-Y., Mukhiddinov M. (2023). Data Anomaly Detection for Structural Health Monitoring Based on a Convolutional Neural Network. Sensors.

[15] Soleimani-Babakamali M.H., Sepasdar R., Nasrollahzadeh K., Sarlo R. (2021). System-Reliability Based Multi-Ensemble of GAN and One-Class Joint Gaussian Distributions for Unsupervised Real-Time Structural Health Monitoring. arXiv.

[16] Boccagna R., Bottini M., Petracca M., Amelio A., Camata G. (2023). Unsupervised Deep Learning for Structural Health Monitoring. Big Data Cogn. Comput..

[17] Byfield R., Shabaka A., Vargas M.M., Tansel I. (2025). Inspection of Additively Manufactured Structures Using Combined Nondestructive Testing (NDT) and Structural Health Monitoring (SHM) Systems. J. Vib. Eng. Technol..

[18] Pathak I., Jha I., Sadana A., Bhowmik B. (2023). CNN-Based Structural Damage Detection Using Time-Series Sensor Data. arXiv.

[19] De Oliveira M.A., Monteiro A.V., Vieira Filho J. (2018). A New Structural Health Monitoring Strategy Based on PZT Sensors and Convolutional Neural Network. Sensors.

[20] Cha Y.-J., Ali R., Lewis J., Büyüköztürk O. (2024). Deep Learning-Based Structural Health Monitoring. Autom. Constr..

[21] Azad M.M., Raouf I., Sohail M., Kim H.S. (2024). Structural Health Monitoring of Laminated Composites Using Lightweight Transfer Learning. Machines.

[22] Modir A., Tansel I. (2022). Analysis of Force Sensing Accuracy by Using SHM Methods on Conventionally Manufactured and Additively Manufactured Small Polymer Parts. Polymers.

[23] Modir A., Tansel I. (2022). Structural Health Monitoring of Additively Manufactured Parts by Combining Infill Design, Multiple Pulse Width Excitation (MPWE), and Deep Learning. J. Vib. Eng. Technol..

[24] Song X., Li D., Cho C. (2024). Image-Based Machine Learning Approach for Structural Damage Detection through Wavelet Transforms. Urban Lifeline.

[25] Avci O., Abdeljaber O., Kiranyaz S., Inman D.J. (2017). Structural Damage Detection in Real Time: Implementation of 1D Convolutional Neural Networks for SHM Applications. Structural Health Monitoring & Damage Detection.

[26] Seventekidis P., Giagopoulos D., Arailopoulos A., Markogiannaki O. (2020). Structural Health Monitoring Using Deep Learning with Optimal Finite Element Model Generated Data. Mech. Syst. Signal Process..

[27] Shi J.Y., Spencer B.F., Chen S.S. (2018). Damage Detection in Shear Buildings Using Different Estimated Curvature. Struct. Control Health Monit..

[28] McLachlan G., Peel D. (2000). Finite Mixture Models.

[29] Bishop C.M. (2006). Pattern Recognition and Machine Learning.

[30] Garrido J., Vales J., Silva-Muñiz D., Riveiro E., López-Matencio P., Rivera-Andrade J. (2025). Adaptive Gaussian Mixture Models-Based Anomaly Detection for Under-Constrained Cable-Driven Parallel Robots. Robotics.

[31] Dempster A.P., Laird N.M., Rubin D.B. (1977). Maximum Likelihood from Incomplete Data via the EM Algorithm. J. R. Stat. Soc. Ser. B.

[32] Ghahramani Z., Jordan M.I. (1996). Supervised Learning from Incomplete Data via an EM Approach. Advances in Neural Information Processing Systems 6.

[33] Schwarz G. (1978). Estimating the Dimension of a Model. Ann. Stat..

[34] Kass R.E., Wasserman L. (1995). A Reference Bayesian Test for Nested Hypotheses and Its Relationship to the Schwarz Criterion. J. Am. Stat. Assoc..

[35] Lee T., Jin S.-S., Kim S.T., Min J. (2025). Online Anomaly Detection for Long-Term Structural Health Monitoring of Caisson Quay Walls. Eng. Struct..

[36] Sawant S., Patil S., Thalapil J., Banerjee S., Tallur S. (2021). Environmental Variation–Compensated Damage Classification and Localization in Ultrasonic Guided Wave SHM Using Self-Learnt Features and Gaussian Mixture Models. arXiv.

[37] Li S., Xin J., Jiang Y., Yang C., Wang X., Ran B. (2024). A Novel Hybrid Model for Bridge Dynamic Early Warning Using LSTM–EM–GMM. Adv. Bridge Eng..

[38] Figueiredo E., Peres N., Moldovan I., Nasr A. (2024). Impact of Climate Change on Long-Term Damage Detection for Structural Health Monitoring of Bridges. Struct. Health Monit..

[39] Xu D., Xu X., Forde M.C., Caballero A. (2023). Concrete and Steel Bridge Structural Health Monitoring—Insight into Choices for Machine Learning Applications. Constr. Build. Mater..

[40] Sonbul O.S., Rashid M. (2023). Algorithms and Techniques for the Structural Health Monitoring of Bridges: Systematic Literature Review. Sensors.

[41] Rasul M. Application of Convolutional Neural Network–Gaussian Mixture Model for Ambient Vibration-Based Structural Damage Detection. Proceedings of the Architectural Institute of Japan Annual Conference.

[42] Farrar C.R., Worden K. (2012). Structural Health Monitoring: A Machine Learning Perspective.

[43] Mammeri S., Barros B., Conde-Carnero B., Riveiro B. (2025). From Traditional Damage Detection Methods to Physics-Informed Machine Learning in Bridges: A Review. Eng. Struct..

[44] Qing X., Liao Y., Wang Y., Chen B., Zhang F., Wang Y. (2022). Machine Learning-Based Quantitative Damage Monitoring for Composite Structures. Int. J. Smart Nano Mater..

[45] Eltouny K. (2023). Unsupervised Learning Methods for Data-Driven Vibration-Based Structural Health Monitoring: A Review. Sensors.

[46] Verma A., Samdani K., Shafi M. (2025). Multimodal Real-Time Anomaly Detection and Industrial Applications. arXiv.

[47] Galati M., Minetola P. (2019). Analysis of Density, Roughness, and Accuracy of the Atomic Diffusion Additive Manufacturing (ADAM) Process for Metal Parts. Materials.

